# Atopy and Inhaled Corticosteroid Use Associate with Fewer IL-17^+^ Cells in Asthmatic Airways

**DOI:** 10.1371/journal.pone.0161433

**Published:** 2016-08-23

**Authors:** Fatemeh Fattahi, Corry-Anke Brandsma, Monique Lodewijk, Marjan Reinders-Luinge, Dirkje S. Postma, Wim Timens, Machteld N. Hylkema, Nick H. T. ten Hacken

**Affiliations:** 1 University of Groningen, University Medical Center Groningen, Department of Pulmonology, Groningen, the Netherlands; 2 University of Groningen, University Medical Center Groningen, Department of Pathology and Medical Biology, Groningen, the Netherlands; 3 University of Groningen, University Medical Center Groningen, Research Institute for Asthma and COPD (GRIAC), Groningen, the Netherlands; 4 Immunology, Asthma and Allergy Research Institute, Tehran University of Medical Sciences, Tehran, Iran; National and Kapodistrian University of Athens, GREECE

## Abstract

**Background:**

Interleukin (IL)-17 plays a critical role in numerous immune and inflammatory responses and was recently suggested to contribute to the pathogenesis of nonatopic (non-eosinophil/neutrophil-dominant) asthma. We aimed to compare expression of IL-17 in bronchial airways between atopic and nonatopic asthmatics, with/without inhaled corticosteroid (ICS) use and to identify its major cellular source.

**Methods:**

Bronchial biopsies from 114 patients with mild-to-moderate asthma were investigated: 33 nonatopic, 63 non-corticosteroid users, 90 nonsmokers. IL-17 expression was correlated with atopy and inflammatory cell counts (EPX, NP57, CD3, CD4, CD8, CD20, CD68), taking ICS use and smoking into account. Multiple linear regression analyses were used to determine the independent factors as well as the most relevant inflammatory cells contributing to IL-17 expression. Double immunostainings were performed to confirm the major cellular source of IL-17.

**Results:**

In non-ICS users, nonatopic asthmatics had more IL-17^+^ cells in the airway wall than atopic asthmatics. In both atopic and nonatopic asthmatics, ICS use was associated with lower numbers of IL-17^+^ cells, independent of smoking. The number of IL-17^+^ cells was associated with the number of neutrophils (B: 0.26, 95% CI: 0.17–0.35) and eosinophils (B: 0.18, 95% CI: 0.07–0.29). The majority of IL-17^+^ cells were neutrophils, as confirmed by double immunostaining.

**Conclusions:**

We show for the first time that atopy and ICS use are associated with lower numbers of IL-17^+^ cells in asthmatic airways. Importantly, IL-17^+^ cells were mostly neutrophils which conflicts with the paradigm that lymphocytes (Th17) are the main source of IL-17.

## Introduction

Asthma is a chronic inflammatory disease of the airways, characterized by reversible airway obstruction and bronchial hyperresponsiveness (BHR) [1]. One of the oldest ways to discern asthmatic patients is based on the presence or absence of atopy [2]. Not surprisingly, the underlying airway pathology of atopic versus nonatopic asthma is different, showing high numbers of eosinophils, T lymphocytes and Th2 cytokines (interleukin (IL)-4 and IL-5) in atopic asthma versus high numbers of neutrophils and non-Th2 cytokines (IL-8) in nonatopic asthma [3]. One of the cytokines that was recently suggested to contribute to the pathogenesis of nonatopic (non-eosinophil/neutrophil-dominant) asthma is IL-17 [4].

IL-17, also called IL-17A, is a proinflammatory cytokine, implicated in the development of autoimmunity, tumorigenesis and host defenses against bacterial and fungal infections [5]. In the lung, increased levels of IL-17 have been demonstrated in inflammatory disorders like asthma and chronic obstructive pulmonary disease (COPD) [6–10]. IL-17 was first shown to be produced by activated CD4^+^ memory T cells [11]. Thereafter, a specific subset of Th cells, namely the Th17 cells, has been put forward as its main producer [12, 13]. Th17 cells have been shown to mediate airway inflammation and hyperresponsiveness associated with non-eosinophilic asthma in mice, and importantly do not respond well to glucocorticoid treatment [14]. In humans, Th17 cells have also been suggested to play a role in regulating a neutrophil and macrophage dominant type of inflammation in the lung, particularly in severe, steroid-insensitive asthma and COPD [6]. In line with this, IL-17 levels were found to correlate positively with sputum neutrophilia in severe asthma [7, 15].

On the other hand, IL-17 has also been implicated in Th2 responses. In mouse models of asthma, Th17 cells were shown to home to the lung and enhance not only neutrophilic airway inflammation but also Th2 cell-mediated eosinophilic airway inflammation [16]. And in patients with allergic asthma increased levels of IL-17 were demonstrated after a challenge with house dust mite [17].

Although there has been substantial interest in elucidating the role of IL-17 in neutrophil-dominant/nonatopic asthma in humans [4, 18], our understanding regarding this phenotype of asthma is still very limited. Although recent studies suggest that a higher level of IL-17 expression is associated with severe asthma, the atopic status was not included in their analysis [19–22]. In fact, there is no data comparing IL-17 expression between atopic and nonatopic asthma patients. We therefore investigated the expression of IL-17 in bronchial biopsies from a large cohort of well characterized atopic and nonatopic asthmatic patients, also taking into account the effect of inhaled corticosteroid (ICS) and smoking. Additionally, we identified the major cellular source of IL-17 in the airway walls of these asthma patients.

## Materials and Methods

### Subjects

We investigated 114 stable, mild-to-moderate subjects with current asthma from our large asthma cohorts that were recruited previously by our research group in the University Medical Center Groningen [23]. Atopic and nonatopic patients, with or without ICS use, aged between 19–71 years were included (Table 1). All patients had a doctor’s diagnosis of asthma and demonstrated reversibility and BHR to histamine and/or adenosine 5’-monophosphate (AMP) [23]. All patients also had alveolar and bronchial exhaled nitric oxide (NO) values on the Aerocrine NO system (Niox; Aerocrine AB, Stockholm, Sweden) measured in accordance with international guidelines as described in an earlier study [23]. Atopic status was determined by Phadiatop for all 114 patients using the ImmunoCap system (Phadia AB, Uppsala, Sweden), and expressed as ratios (fluorescence of the serum of interest divided by the fluorescence of a control serum). A positive Phadiatop was defined as patient serum/control serum >1. The Medical Ethics Committee of the University Medical Center Groningen approved the study protocol and all subjects gave written informed consent.

**Table 1:** Patient characteristics.

	ICS user		Non-ICS user	
	*Atopic (n = 39)*	*Nonatopic (n = 12)*	*Atopic (n = 42)*	*Nonatopic (n = 21)*
**Sex, males/females**	19/20	6/6	26/16	10/11
**Age, Years**	50 (22–68)	50 (30–71)	48 (22–70)	50 (21–65)
**BMI, Kg/m**^**2**^	25.6 (19–39)	26.3 (19.5–44.2)	27.2 (19.3–40.4)	27.2 (21.4–42.4)
**Smoking, yes/no**	6/33	1/11	12/30	5/16
**Age of asthma onset**	8 (1–55)	21 (1–40)	7 (1–48)	14.5 (3–22)
**Asthma duration**	44 (3–58)	22 (5–44)	42 (4–60)	35.5 (1–57)
**ICS dose, μg/day**	800 (28–2000)	1000 (400–2000)	____	____
**High doses of ICS (>1000μg/day)**	9 (23.1%)	5 (41.7%)	____	____
**FEV**_**1**_**, % pred**	95.6 (42.5–121.3)	104.5 (52.1–135.3)	97.9 (59.8–122.0)	105.4 (84.0–122.7)^#^
**FEV**_**1**_**/VC, %**	71.5 (39.9–96.7)	73.3 (39.4–94.1)	71.7 (47.6–97.7)	80.0 (67.0–93.6)*
**AMP PC20, mg/ml**	51.5 (0.0–640)	640 (0.01–640)	83.9 (0.02–640)	640 (0.08–640)^#^
**Reversibility, % pred**	8.7 (-0.8–38.4)	7.3 (0.73–20.0)	9.1 (-0.24–28.7)	6.2 (-1.4–17.4)
**Total IgE (IU/ml)**	146 (5–1668)	78 (17–359)	94 (9–1302)	36.5 (1–295)*
**Specific IgE (PAU/l)**	24.4 (1.7–128)	0.21 (0.15–0.92)*	22.5 (1.2–106)	0.26 (0.06–0.76)*
**NO Bronchial (nl/s)**	0.89 (0.2–10.4)	0.3 (0.09–2.5)*	0.8 (0.06–3.2)	0.5 (0.09–1.1)
**NO Alveolar (ppb)**	6.1 (1.5–51.7)	4.3 (1.9–11.9)	5.9 (2.08–18.3)	3.8 (0.9–8.2)*

Definition of abbreviations: ICS: inhaled corticosteroid (beclomethasone equivalent); FEV_1_: forced expiratory volume in one second, measured after inhalation of 800 μg albuterol; VC: vital capacity; MEF_50_: maximum expiratory flow rate at 50% of vital capacity; AMP PC_20_: provocative concentration of adenosine 5'- monophosphate causing a 20% fall in FEV_1_; All values was obtained 15 min after inhalation of 1 mg terbutaline. Reversibility: change in FEV_1_, expressed as increase in percentage predicted normal value after 400 μg of albuterol. Normal reversibility was defined a greater than 12% and 200 ml increase in FEV_1_ following inhalation of the bronchodilator (Global Initiative for Asthma. Global Strategy for Asthma Management and Prevention. 2014. Available from: http://www.ginaasthma.com). PAU/l: Phadia Arbitrary Units per litre; NO: nitric oxide

Values are number (no.) or medians with minimum-maximum ranges in parentheses.

*p<0.05 vs atopic (in ICS or non-ICS user);

^#^trend: 0.05<p<0.10 vs atopic (in ICS or non-ICS user).

### Immunohistochemical staining and cellular quantification of bronchial biopsies

Paraffin embedded bronchial biopsies were cut into 3-μm-thick sections. Sections were deparaffinized and, after antigen retrieval, incubated with appropriate polyclonal antibodies against IL-17 (R&D Systems, polyclonal Goat anti-Human, AF-317-NA), using the DAKO autostainer in three consecutive runs. The slides were included in random fashion in each run to avoid group wise staining ^(19)^. The number of positive cells was counted by a blinded observer in the submucosal area 100 μm under the basement membrane in the biopsy sample ^(19)^ using Aperio Image Scope software. The same techniques had been already applied for immunohistochemical staining and cellular quantification of other inflammatory cells including: neutrophils (NP57, DAKO, Glostrup, Denmark), eosinophils (eosinophil peroxidase; EPX, laboratories of NA Lee and JJ Lee, Mayo Clinic, Scottsdale, AZ), macrophages (CD68, DAKO, Glostrup, Denmark), mast cells (AA1, DAKO, Glostrup, Denmark) and T-cells (CD3, CD4, CD8, DAKO, Glostrup, Denmark)[23].

Double immunostainings were performed to elucidate whether granulocytes are a source of IL-17 in bronchial biopsies of asthmatics. Primary neutrophil and eosinophil antibodies suitable for double staining with IL-17 were used; a polyclonal Rabbit anti-Human Myeloperoxidase (MPO) antibody (DAKO, Glostrup, Denmark) was used to identify neutrophils and a Mouse anti-Human EPX antibody (Mayo Clinic, Scottsdale, AZ, USA) to identify eosinophils. After deparaffinizing the slides, a heat-induced antigen (epitope) retrieval protocol was used and blocking for endogenous peroxidase was applied. As secondary antibodies, peroxidase conjugated Swine anti-Rabbit IgG Antibody (DAKO, Glostrup, Denmark) was used for detecting MPO stained cells, biotinylated labeled Rabbit anti-Mouse antibody (DAKO, Glostrup, Denmark) for detecting EPX stained cells and Alkaline Phosphatase conjugated Donkey anti-Goat IgG antibody (SouthernBiotech, USA) for detecting IL-17 stained cells. Double immunostaining with lymphocytes was unnecessary because the vast majority of the IL-17^+^ cells showed the morphology of granulocytes. This was confirmed by the MPO/IL17 and EPX/IL17 double immunostainings.

### Statistics

All analyses were performed using SPSS software (version 19.0; SPSS Inc., Chicago, IL). Normality of distributions was assessed using histograms and/or p-p plots.

For quantitative variables analysis, one-way ANOVA followed by Tukey post-hoc test was performed for multiple comparisons and t tests or Mann-Whitney U tests was used for two samples comparison.

Chi-square tests were used to compare groups for dichotomous variables. Correlations were evaluated by Pearson (for normally distributed data) or Spearman (for non-normally distributed data) tests. Multiple linear regression analysis was used to assess the independent contribution of ICS use (yes/no), smoking (smoking vs. nonsmoking) and Phadiatop (atopic vs. nonatopic) to IL-17 expression (dependent variable). To find the most relevant inflammatory cells contributing to IL-17 expression, additional linear regressions were performed on inflammatory markers (neutrophil, eosinophil, T-cell, macrophage, mast cell) as independent variables (separately for each one or in combination with other inflammatory cells) and IL-17 as dependent variable, adjusting for atopy, smoking status and ICS use. For all statistical analyses, p values <0.05 were considered statistically significant.

## Results

### Inflammatory cells counts in blood, sputum and bronchial biopsies

In the group of asthma patients who did not use ICS, there were trends towards lower blood eosinophil counts (p = 0.08) and lower percentage of sputum eosinophils (p = 0.06) in nonatopic asthma patients compared to atopic patients (Table 2). In addition, nonatopic patients had more neutrophils in the bronchial submucosa than atopic patients, whereas the atopic asthmatics had more eosinophils (Table 2). In the group of patients who did use ICS, the nonatopic individuals had more CD8^+^ cells in the bronchial submucosa than the atopic ones (Table 2).

**Table 2:** Inflammation in atopic and nonatopic asthmatics.

	ICS user		Non-ICS user	
	*Atopic (n = 39)*	*Nonatopic (n = 12)*	*Atopic (n = 42)*	*Nonatopic (n = 21)*
**Blood**				
Eosinophil, ×10^9^/L	0.21 (0.04–0.97)	0.15 (0.07–0.78)	0.18 (0.02–0.5)	0.12 (0.03–0.46)^#^
**Sputum**				
Total cells, ×10^6^/ml	0.5 (0.1–5.2)	0.5 (0.15–1.8)	0.3 (0.3–2.1)	0.4 (0.1–2.2)
Eosinophil, %	1.2 (0–67.1)	0.37 (0–38.6)	1.02 (0–16.7)	0.2 (0–5.2)^#^
Neutrophil, %	55.8 (16.8–88.8)	53.4 (43.7–88.6)	59.6 (19.8–93.6)	57 (20.7–94.5)
**Bronchial biopsies (/0.1mm**^**2**^**)**				
Eosinophils (EPX^+^)	1.1 (0–32.4)	1.8 (0–23.3)	2.6 (0–40.3)	0.3 (0–19.1)*
Neutrophils (NP57^+^)	4.8 (0–33.8)	6.2 (0.9–16.2)	5.8 (0–38.2)	10.4 (0–46)*
Mast cells (AA1^+^)	8 (0–22.2)	8.3 (0–16.7)	8.1 (0–26.3)	8.7 (0–24.4)
Macrophages (CD68^+^)	11.8 (0–37.1)	19.5 (4.3–57)	11.6 (0.31–30.1)	13.2 (0–36.5)
T lymphocytes (CD3^+^)	65 (4.2–219)	81.1 (29.7–216)	77.3 (12.5–294)	61 (18–136.7)
T lymphocytes (CD4^+^)	14.3 (0–101.2)	27.4 (4.3–57.6)	22 (0–67.2)	18.5 (0.85–58.2)
T lymphocytes (CD8^+^)	17.7 (0.98–205.2)	38.3 (0–78.4)*	21.1 (1.02–112.3)	15.9 (3–139)
B lymphocytes (CD20^+^)	2.9 (0–37.1)	6.05 (0.9–55.5)^#^	1.9 (0–98.3)	2.6 (0–20.5)
IL-17^+^ cells	6.2 (1.2–18.4)	8.5 (4.6–16.9)	11.5 (3.9–29.6)	15.3 (9.7–24.9)*

Values are medians with minimum-maximum ranges in parentheses.

*p<0.05 vs atopic (in ICS or non-ICS user);

^#^trend: 0.05<p<0.10 vs atopic (in ICS or non-ICS user).

### Lower IL-17 expression in bronchial biopsies associated with atopy and ICS use

**Fig 1:**
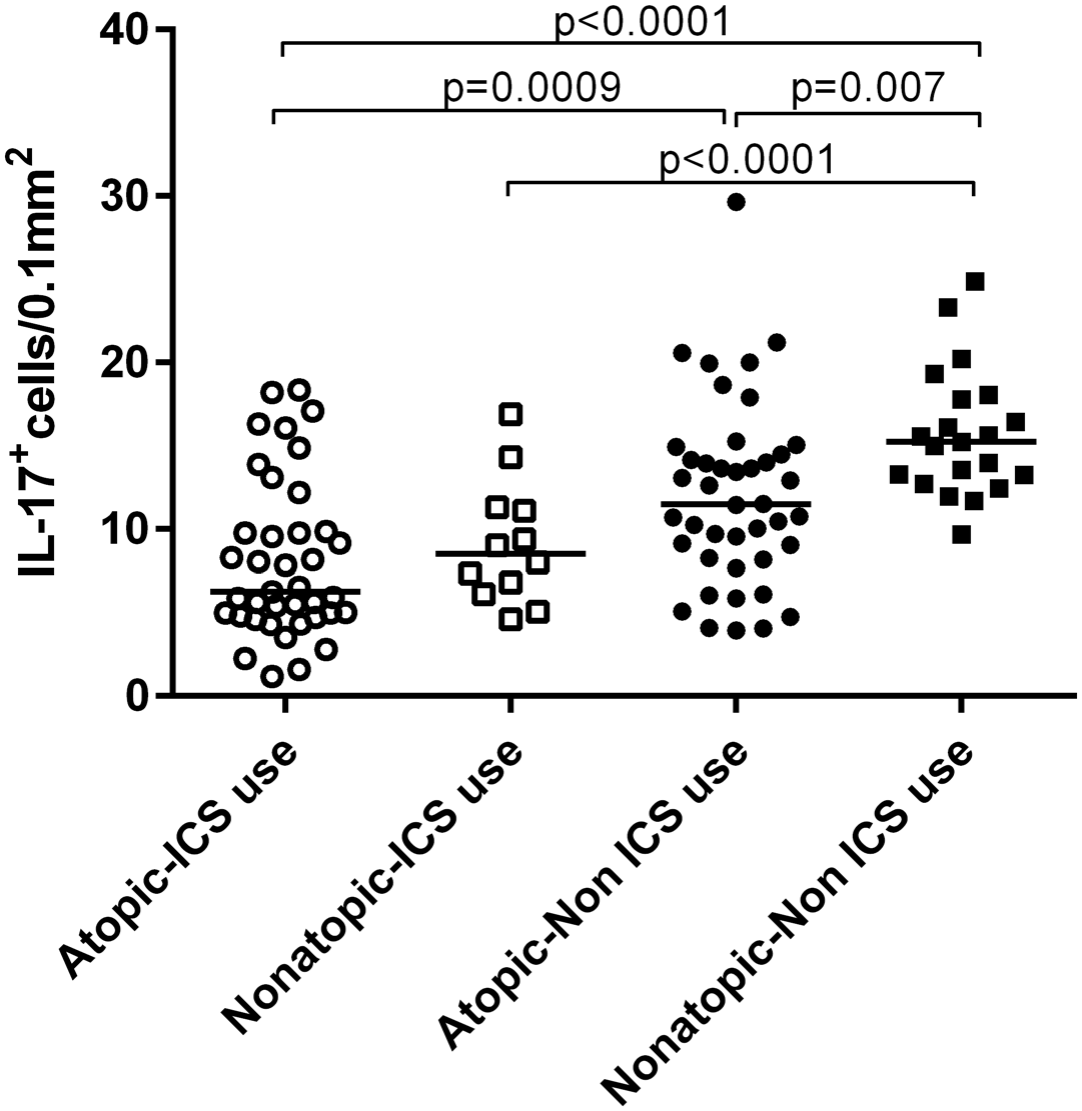
Number of IL-17^+^ cells in submucosa in bronchial biopsies from atopic and nonatopic asthmatics who are inhaled corticosteroid (ICS) users or non-ICS users.

**Fig 2:**
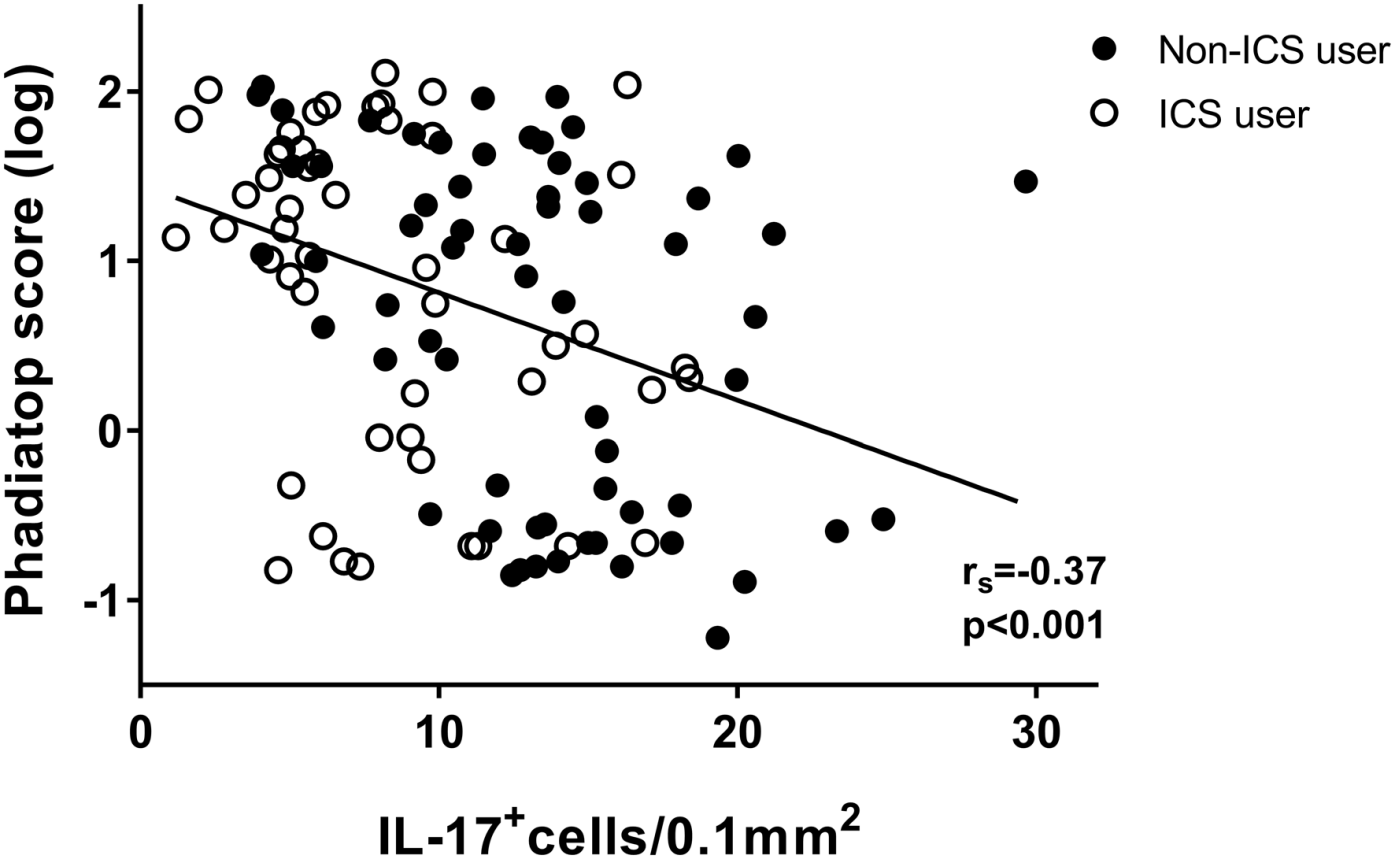
Negative correlation between the number of IL-17^+^ cells in the submucosa of bronchial biopsies and serum specific IgE (Phadiatop) from asthmatics (rs = -0.37; P<0.001).

In the group of non-ICS users, there were significantly more IL-17^+^ cells in the bronchial submucosa of nonatopic asthmatics compared to atopic ones (Fig 1). In line with this finding, a negative correlation was found between IL-17^+^ cells numbers and the Phadiatop score (r_s_ = -0.37, p<0.001) (Fig 2).

Both atopic and nonatopic asthma patients treated with ICS had lower numbers of IL-17^+^ cells than those without ICS treatment (Fig 1). There were 9 subjects in the atopic group and 5 subjects in the nonatopic group who used high doses of ICS (>1000ug daily) (Table 1). There was no association between doses of ICS (high doses vs. mild-moderate doses) and cellular infiltrate.

There was a significant negative correlation between IL-17^+^ cells numbers and reversibility levels in the total population who did not use ICS (both atopic and nonatopic subjects) (r_s_ = -0.33; p = 0.01) (S1A Fig) in line with a negative correlation between neutrophils levels and reversibility levels in the total population who used ICS (both atopic and nonatopic subjects) (r_s_ = -0.27; p = 0.04). There was also a negative correlation between FEV_1_% predicted and IL-17 ^+^ cells numbers in the atopic individuals who did not use ICS (r_s_ = -0.39; p = 0.01)(S1B Fig).

There was no association between current smoking and IL-17 levels (S2 Fig) and current smoking had no effect on IL-17 counts in all groups of asthmatics.

Using regression analyses, we demonstrated that the absence of atopy (B: -2.42, 95% CI: -4.16- -0.69) and non-ICS use (B: -4.29, 95% CI: -5.85- -2.74) most strongly contributed to the number of IL-17^+^ cells. There was no significant contribution of smoking status (defined as smoking and nonsmoking) (B: 0.17, 95% CI: -1.78–2.14).

### IL-17 expression positively associated with neutrophilic inflammation

**Fig 3:**
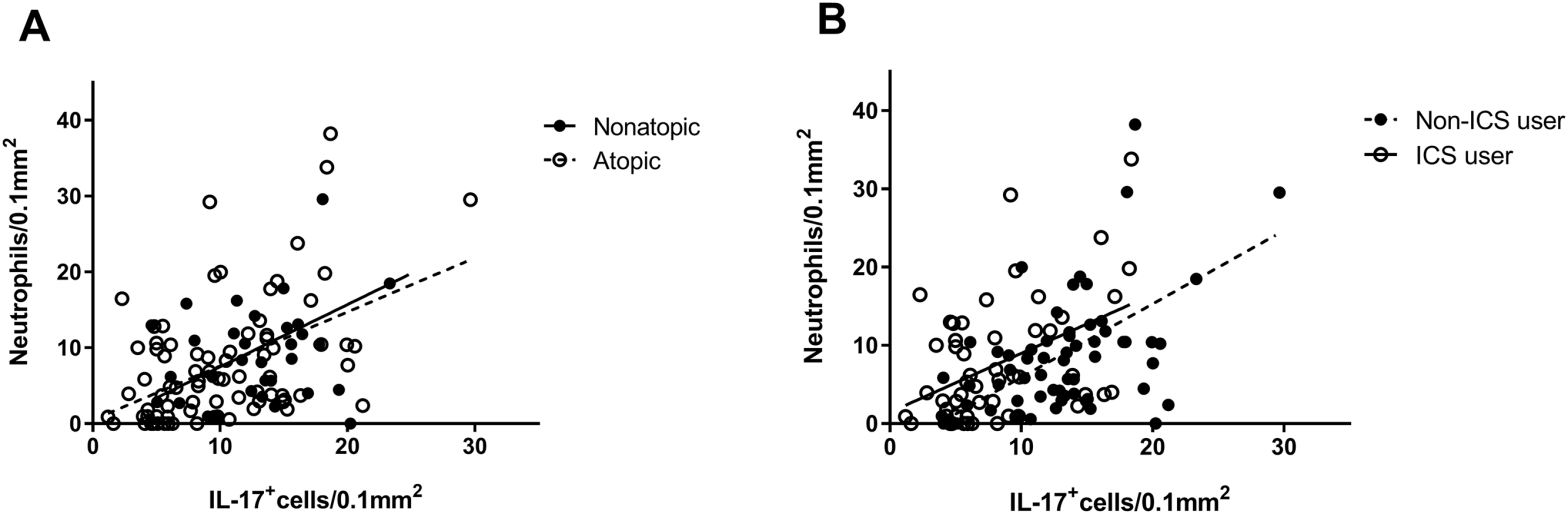
Positive correlation between the number of IL-17^+^ cells and neutrophils in the submucosa of bronchial biopsies from atopic (r_s_ = 0.44; p<0.001) and nonatopic (r_s_ = 0.45, p = 0.009) asthmatics (A), or from asthmatics who are inhaled corticosteroid (ICS) (r_s_ = 0.35; p = 0.01) and non-ICS (r_s_ = 0.48; p<0.0001) users (B).

The number of IL-17^+^ cells in airway wall biopsies correlated significantly with the number of neutrophils, both in atopic (r_s_ = 0.44; p<0.001) and nonatopic asthmatics (r_s_ = 0.45; p = 0.009) (Fig 3A), and both in ICS users (r_s_ = 0.35; p = 0.01) and non-ICS users ((r_s_ = 0.48; p<0.0001)(Fig 3B). Additionally, in atopic asthmatics the number of IL-17^+^ cells correlated significantly with the number of eosinophils (r_s_ = 0.36; p = 0.001), CD4^+^ cells (r_s_ = 0.33; p = 0.003), CD3^+^ cells (r_s_ = 0.31; p = 0.005), and CD8^+^ cells (r_s_ = 0.27; p = 0.015).

Using regression analysis and after adjusting for atopy, ICS use and smoking, we confirmed the contribution of neutrophils (B: 0.26, 95% CI: 0.17–0.35) as well as eosinophils, with lower value (B: 0.18, 95% CI: 0.07–0.29) to the number of IL-17^+^ cells. Other inflammatory cells did not contribute significantly to IL-17 expression (data not shown).

**Fig 4:**
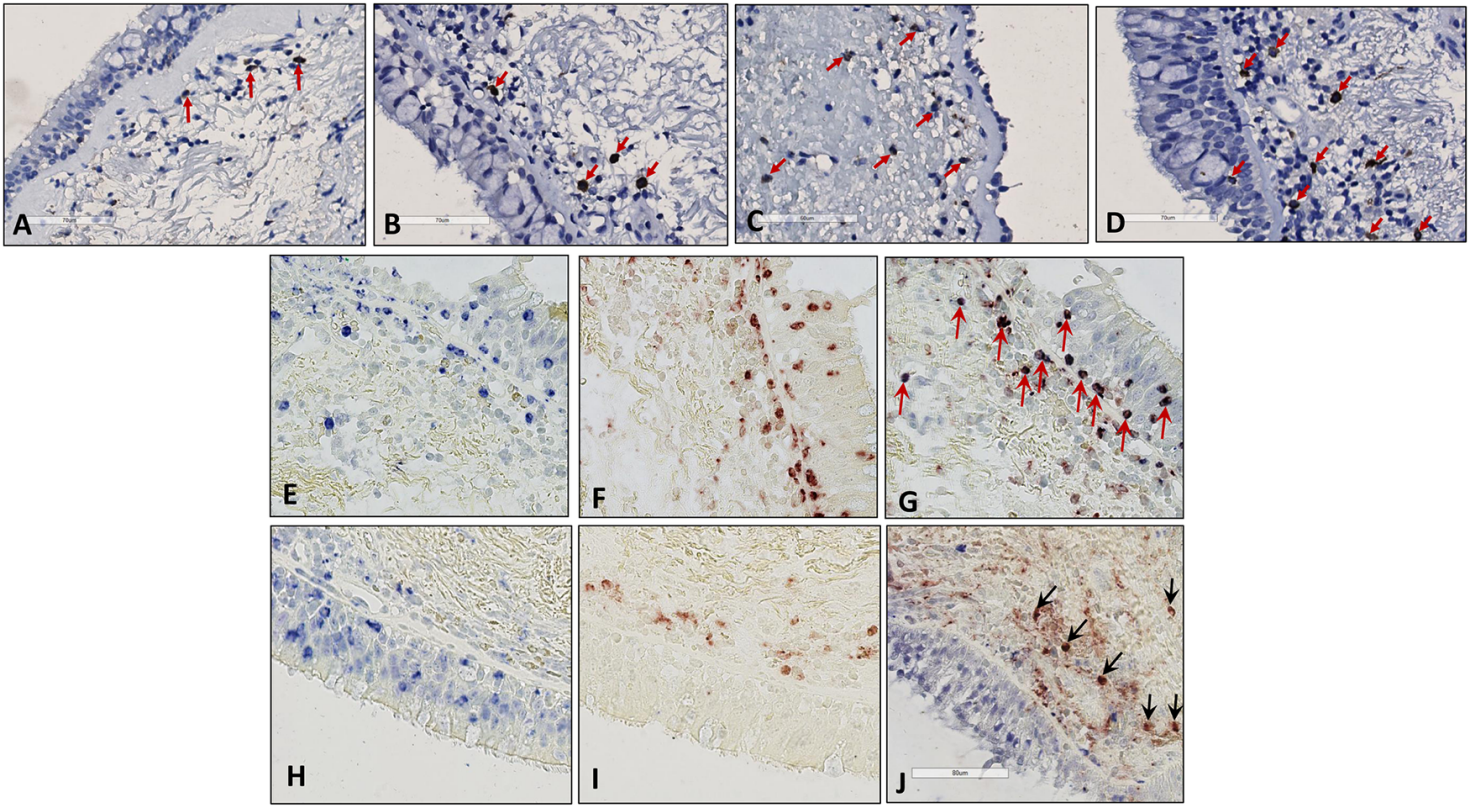
IL-17 expression in the submucosa of bronchial biopsies of 4 groups of studied population. atopic inhaled corticosteroid (ICS) user (frame A), nonatopic ICS user (frame B), atopic non-ICS user (frame C), nonatopic non-ICS user (frame D). Single staining for IL-17 (frame E; blue) and MPO (frame F; red) and double staining for IL-17 and MPO (frame G; purple) in adjacent sections of a nonatopic non-ICS user asthmatic patient. Single staining for IL-17 (frame H; blue) and EPX (frame I; red) and double staining for IL-17 and EPX (frame J; purple) in adjacent sections of an atopic non-ICS user asthmatic patient.

We found that the majority (~90%) of IL-17^+^ cells were granulocytes, mostly neutrophils, as indicated by double staining for IL-17 and MPO and nuclear morphology (Fig 4). In addition, we identified a few IL-17^+^ eosinophils, as indicated by double staining for IL-17 and EPX. Fig 4 shows one representative IHC staining for IL-17 for all 4 studied subgroups (frame A-D) and double staining of IL-17 and MPO (frame E-G) and staining of IL-17 and EPX (frame H-J) from an asthmatic patient with high numbers of neutrophils (frame E-G) or eosinophils (frame H-J).

## Discussion

This is the first study comparing cellular IL-17 expression in well characterized atopic and nonatopic asthma patients. We demonstrate that IL-17 was particularly expressed by neutrophils in the airway biopsies, contrasting with the paradigm that lymphocytes (Th17) are the main source of IL-17. Our results show that in patients who do not use ICS, nonatopic asthmatics have more IL-17 expressing cells in the airway wall than atopic asthmatics. In contrast, ICS use was associated with lower numbers of IL-17 expressing cells in both atopic and nonatopic asthmatics.

A new finding of our study is that IL-17 expressing cells in bronchial biopsies of asthma patients were predominantly granulocytes and not lymphocytes. We confirmed this by double immunostaining with IL-17 and MPO and by demonstrating a strong positive correlation between IL-17 expressing cells and neutrophils. Although, perhaps surprising, neutrophils have been reported as a source of IL-17 in humans [24, 25] as well as in animal studies [25–29]. In vitro investigations also showed production of IL-17 by stimulated neutrophils with immune complex [27]. Eosinophils may be another source of IL-17, as suggested by double immunostaining of IL-17 and EPX, and by the significant correlation between IL-17^+^ cells and eosinophil numbers in atopic asthma patients. Previous findings in the literature are in line with our finding that IL-17 expressing cells in the airways may be granulocytes. Eustace *et al* showed that IL-17 in bronchial biopsies of COPD patients was expressed by neutrophils, next to mast cells, T cells, and B cells in the subepithelium of the small airways [30]. Molet *et al* demonstrated in asthma that eosinophils in sputum, brochoalveolar lavage fluid, and peripheral blood express IL-17 [10]. Finally, Tan *et al* demonstrated in children with cystic fibrosis that neutrophils and γδT cells in the airways produce IL-17, next to Th17 cells [31]. These data together support the reports showing the early sources of IL-17 are the innate immune cells and they have a central role in the initiation of IL-17-dependent immune responses, even before the first CD4^+^ T cell sees its cognate antigen and initiate the Th17 development program [32].

We found more IL-17 expressing cells in the airway wall of nonatopic than atopic asthmatics, that is those who did not use ICS. It has been suggested that IL-17 may contribute to the pathogenesis of neutrophil-dominant/nonatopic rather than to eosinophil-dominant/atopic asthma [4]. Presence of fewer eosinophils and more neutrophils in our nonatopic asthmatic subjects and the significant contribution of both cell types to IL-17^+^ cells in our biopsies support this hypothesis. Interestingly, we found ICS use to be associated with lower IL-17 expression in bronchial biopsies of both atopic and nonatopic asthmatics. This is in line with a bronchial biopsy study in 10 patients with moderate-to-severe asthma (all atopic) demonstrating a significantly reduction in the number of IL-17^+^ cells in the airways after a 2-week course of oral corticosteroid treatment [8]. Accordingly, IL-17 levels in sputum of 15 mild-moderate and 15 severe asthmatics decreased after one month of ICS treatment [15]. *In vitro* data are also in line with these findings as corticosteroids could inhibit IL-17 induction of cytokines in epithelial cells and fibroblasts [10]. We have also shown before that corticosteroids inhibit IL-17A-induced IL-8 production of epithelial cells [33].

Regarding the effect of IL-17 levels on the lung function we found a negative correlation between FEV_1_% predicted and IL-17 levels in the atopic individuals who did not use ICS. In line with our finding, Irvin *et al* found a negative correlation between FEV_1_% and IL-17 levels in their asthmatic population [20]. Reduced airway patency due to IL-17 mediated airway inflammation may be responsible for this negative association, but also direct sensitization of airway smooth muscle may play a role, as has been suggested in mouse with house-dust mite-induced allergy [34]. Such a direct role of IL-17 in smooth muscle cell contraction is in accordance with findings of a clinical trial demonstrating clinically meaningful effects of anti–IL-17A, especially in a group with high reversibility of FEV_1_ in response to albuterol [35]. However, our study seems to contradict these results as we found an inverse relationship between IL-17 expression and reversibility of FEV_1_ to albuterol. A direct comparison between the two studies is unfortunately not possible, as Busse et al didn't measure expression of IL-17 levels in their studied population [35]. Clearly, more research is necessary to understand the “high IL-17 phenotype” of asthma and its consequences for personalized medicine.

In our study, IL-17 levels was significantly correlated with neutrophilic inflammation but smoking did not contribute to the expression of IL-17. This supports the previous finding by Doe *et al* where IL-17A and IL-17F expression in the submucosa of the lung tissue was not associated with smoking status in their asthmatics [9]. However, our finding contrasts with a study in healthy smokers and COPD patients, showing that smokers have more IL-17 expressing cells in the submucosa than nonsmokers [36]. We conclude that atopy and ICS use may associate with a lower expression of IL-17 and that there are contradictory findings regarding the contribution of smoking. One of the limitations of our study is that the scarce biopsy material did not allow further investigating a potential explanation for the effect of ICS on IL-17^+^ cells. A very recent study shows that IL-17A/IL-4 dual producing cells are important in asthma and may provide a potential explanation for ICS use decreasing IL-17A^+^ cells [20]. Future studies on human biopsy staining are warranted.

## Conclusion

In summary, we here show that the IL-17^+^ cells present in airway wall biopsies of asthmatics are mostly neutrophils and to a smaller extent eosinophils, and not, as the general paradigm assumes lymphocytes (Th17). This is of interest since nonatopic asthmatics who do not use inhaled corticosteroids have higher IL-17 expression in bronchial biopsies than atopic asthmatics, suggesting a potential role of IL-17 in the pathogenesis of nonatopic asthma. ICS use was associated with lower numbers of IL-17^+^ cells in both atopic and nonatopic asthmatics, suggesting a beneficial effect of ICS in general.

## Supporting Information

S1 Fig Negative correlation, in non-inhaled corticosteroid (ICS) users, between the number of IL-17^+^ cells in the submucosa of bronchial biopsies and reversibility (%Predicted) in atopic and nonatopic asthmatics (r_s_ = -0.33; p = 0.01) (A), and with FEV_1_% predicted in atopic asthmatics (r_s_ = -0.39; p = 0.01)(B). Solid circles are nonatopic asthmatics, and open circles are atopic asthmatics.
(TIF)

S2 Fig The number of IL-17^+^ cells in submucosa in bronchial biopsies from smoking and nonsmoking asthmatics, who are inhaled corticosteroid (ICS) users or non-ICS users. Solid circles are non-ICS users, and open circles are ICS users.
(TIF)

## Abbreviations

BHR: Bronchial hyperresponsiveness
IL: Interleukin
COPD: chronic obstructive pulmonary disease
ICS: Inhaled corticosteroid
AMP: Adenosine 5’-monophosphate
MPO: Myeloperoxidase
EPX: Eosinophil peroxidase
r_s_: Spearman rank-order coefficient
FEV_1_: Forced expiratory volume in one second
VC: Vital capacity
MEF_50_: Maximum expiratory flow rate at 50% of vital capacity
AMP PC_20_: Provocative concentration of adenosine 5'- monophosphate causing a 20% fall in FEV_1_
NO: Nitric oxide
