# Supplementary figures and images for: Atopy and Inhaled Corticosteroid Use Associate with Fewer IL-17+ Cells in Asthmatic Airways
Source: PLoS One. 2016 Aug 23;11(8):e0161433. doi: 10.1371/journal.pone.0161433 (PMC4994949; doi:10.1371/journal.pone.0161433)

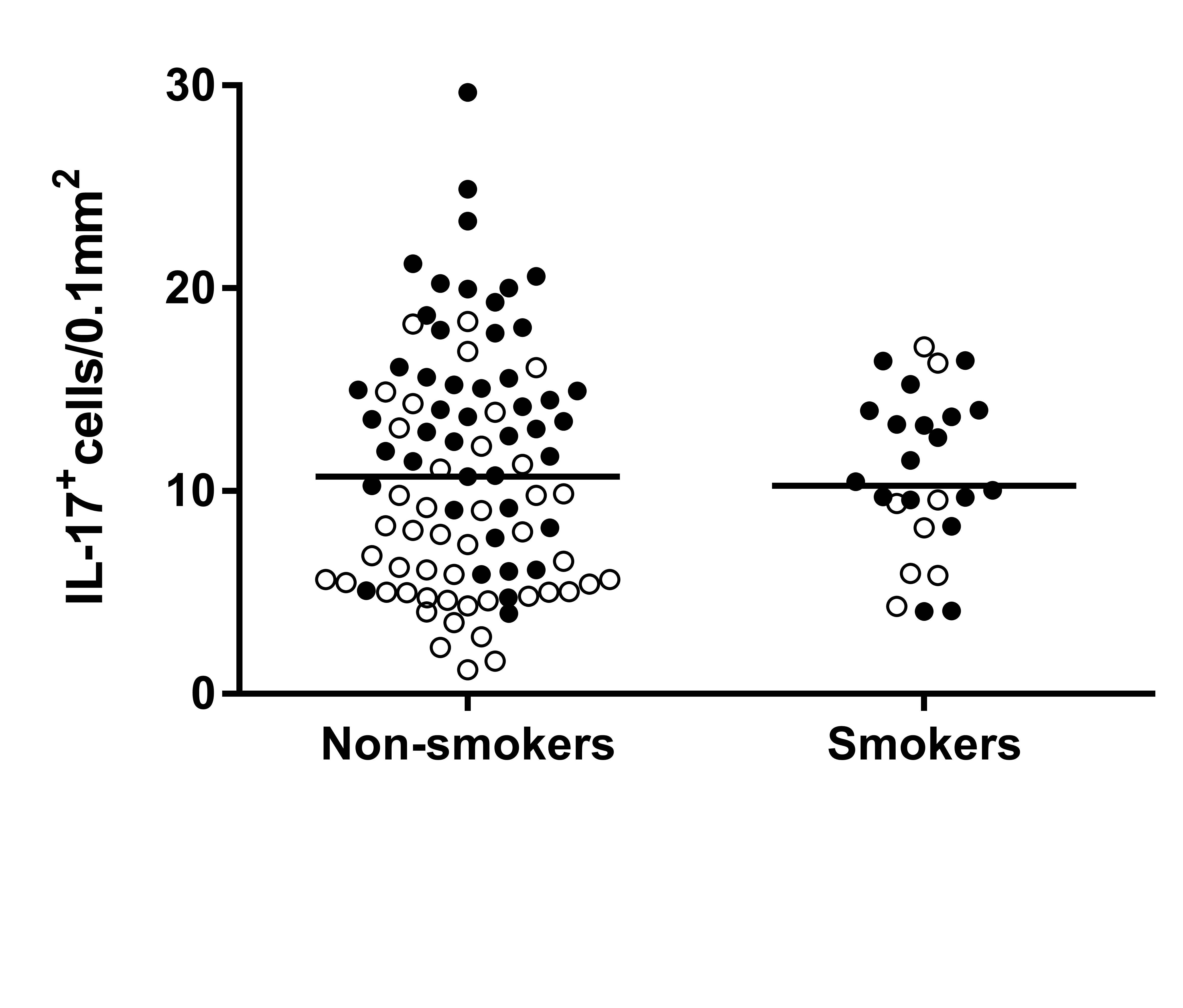

Supplement: S2 Fig — Solid circles are non-ICS users, and open circles are ICS users. (TIF) [file pone.0161433.s002.tif]
